# Differential Effects of Furnidipines’ Metabolites on Reperfusion-Induced Arrhythmias in Rats In Vivo

**DOI:** 10.1371/journal.pone.0089477

**Published:** 2014-02-28

**Authors:** Katarzyna A. Mitrega, Maurycy Porc, Tadeusz F. Krzeminski

**Affiliations:** Chair and Department of Pharmacology, Medical University of Silesia, Katowice, Poland; Virginia Commonwealth University, United States of America

## Abstract

We previously established that furnidipine (FUR) and oxy dihydropyridines prevent rats mortality by strong reduction of the lethal arrhythmias in reperfusion. Therefore we decided to study the influence of three main metabolites (M-2, M-3, M-8) of FUR on ischemia-and reperfusion- induced arrhythmias and hemodynamic parameters in rat model to examine their independent activity. The metabolites (M-2, M-3, M-8) were given orally 20 mg/kg (24 and 1 h before ischemia). Mortality was significantly diminished in M-2 and M-3 treated groups with M-3 preventing animal mortality entirely. All three examined substances significantly reduced the duration and incidence of ventricular fibrillation (VF) with M-3, once again, completely preventing VF. Moreover, only M-3 significantly decreased the duration of ventricular tachycardia but had no influence on their incidence. Through the occlusion and reperfusion periods, M-2 and M-3 were markedly less hypotensive than M-8 and did not influence on heart rate. We conclude that two tested metabolites of FUR, M-3 and M-2 exhibited the most pronounced anti-arrhythmic effect being at the same time the most normotensive and therefore caused the most beneficial effects.

## Introduction

1,4 dihydropiridines (DHP), known as a “privileged structures”, are widely use mainly in hypertension treatment [1], [2], cerebral circulatory disturbances and some forms of angina pectoris [3]. The first synthesized dihydropiridine with proved L-type calcium channel blocking activity was nifedipine (NIF) [4] and after that many others DHP were found as an effective drugs in cardiovascular disorders [5].

In our laboratories we have previously compared four DHP (furnidipine, nifedipine, nimodipine, nitrendipine) given orally or intravenously in ischemia and reperfusion-induced arrhythmias model in rats and we established that the most cardio-protective effect was achieved by furnidipine (FUR) and that the two routes of administration exert various influence on hemodynamic parameters. Furthermore, we have recently published the results of ours study which revealed the differences in effects between DHP and oxy first-pass agents - oxyDHP [6].

Speculating that the different profile of activity between FUR and for example NIF might be caused by the various action of their metabolites, we have decided to study the influence of three metabolites (M-2, M-3, M-8) of FUR on mortality, reperfusion arrhythmias and hemodynamic parameters in the same model to examine their independent activity.

## Materials and Methods

### Animals

Male Sprague – Dawley rats (Central Animal Farm, Medical University of Silesia, Katowice, Poland) weighing approx. 295±5 g and maintained under standard condition (ambient temperature 21–23°C; with 12 h dark/light cycle) with *ad libitum* access to food (standard LSM diet, Motycz, Poland) and tap water, served as experimental animals. The animals were fasted overnight before the experiment. The study was performed with the approval of the Local Bioethical Committee and all experiments were conducted in accordance with NIH regulations of animals care described in the “Guide for the Care and Use of Laboratory Animals” (NIH publication, p. 2–107, revised 1996).

### Drugs and Reagents Used

**Figure 1:**
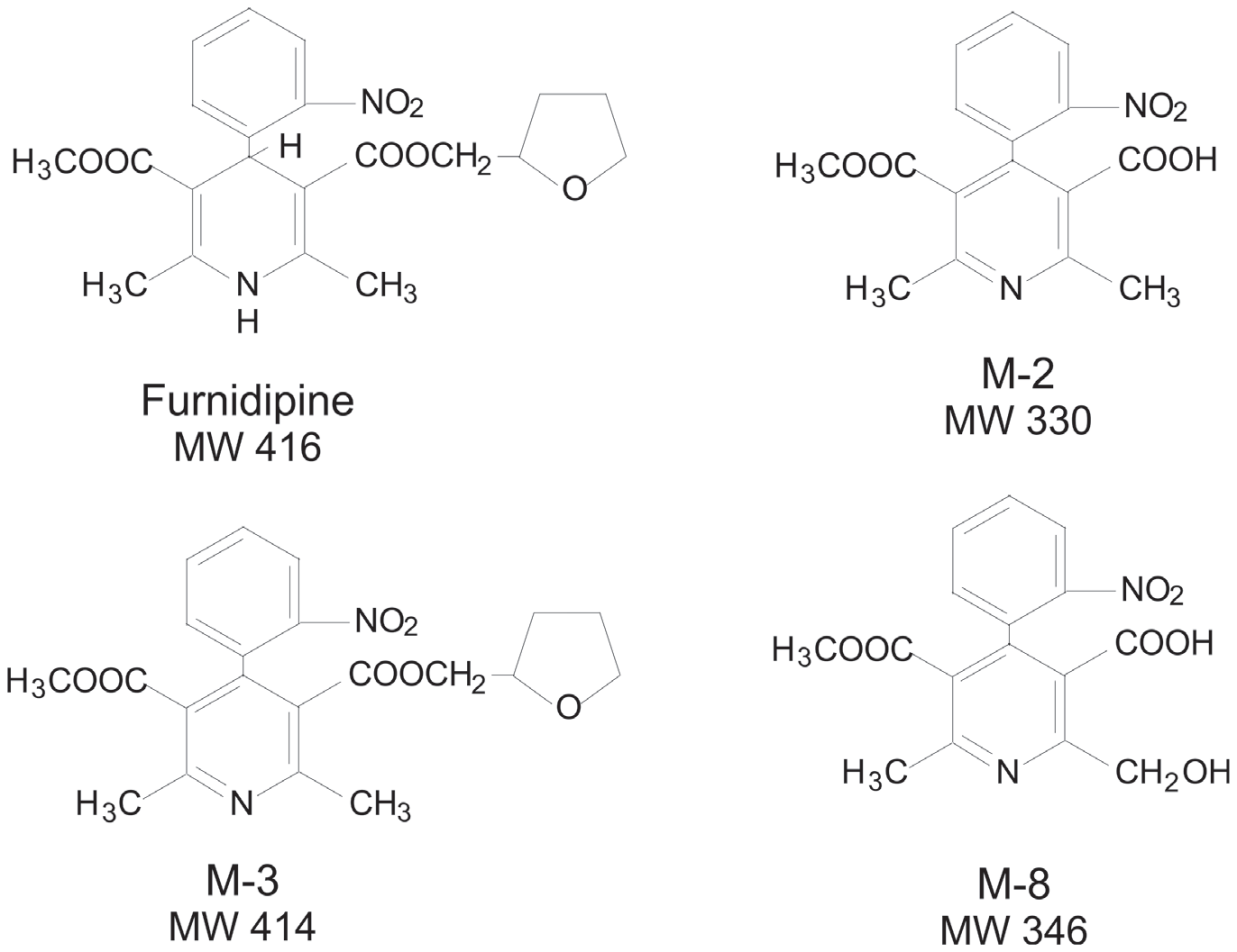
Structure and molecular weight of the three tested major metabolites of furnidipine, M-2, M-3 and M-8.

Three metabolites of furnidipine (M-2, M-3, M-8) were supplied by Cermol S.A., Geneva, Switzerland (Fig. 1). Unless otherwise stated, all other drugs and reagents were of the highest purity and were supplied by Sigma Chemical Co. (Deisenhofen, Germany). For oral administration solutions of furnidipines’ metabolites were prepared in 0.4% aqueous dimethylsulfoxide (DMSO). Proper care was taken while preparing all solutions to avoid photo-decomposition.

### Procedure

For this study an improved preparation previously described by Selye et al. [7] and a modified method of Clark et al. [8] and Crome et al. [9] was used. The complete experimental procedure was performed according to procedures described elsewhere [6], [10]–[17]. The Lambeth Convention was used as a guideline for this research [18].

In brief, the rats were anaesthetized with pentobarbital (60 mg/kg, ip; pentobarbital sodium salt). To compare the depth of anesthesia, reflex response to noise and reflex response to pain induced by the pinching of the limbs and distal portion of the tail, were tested in each rat at the beginning and the end of the experiment as prescribed by Stormant et al. [19] and Weatherall [20]. The left common carotid artery was cannulated with a filled catheter (saline with 2 IU/mL heparin). In the external jugular vein, a PE 50 catheter was placed for the injection of the Evan’s blue dye (to confirm successful reperfusion) and for lethal anesthesia (always pentobarbital) at the end of the experiment. Rectal temperature was maintained at approximately 38°C. The trachea was cannulated to allow artificial ventilation with room air (Rodent VENTILATOR-UB 7025, stroke volume 0.8 mL/100 g body weight and rate 54 strokes/min, Hugo Sachs Elektronik, March-Hugstetten, Germany). The chest was opened by a left thoracotomy. After opening the pericardium, the heart was not exteriorized and a sling (PROLENE 6/0, EH 7245H, Ethicon GmbH, Norderstedt Germany) was placed around the left anterior descending coronary artery (LAD) close to its origin. This modified procedure allowed for reduction of the additional heart damage as well as the mechanically induced arrhythmias development in occlusion, especially.

**Figure 2:**
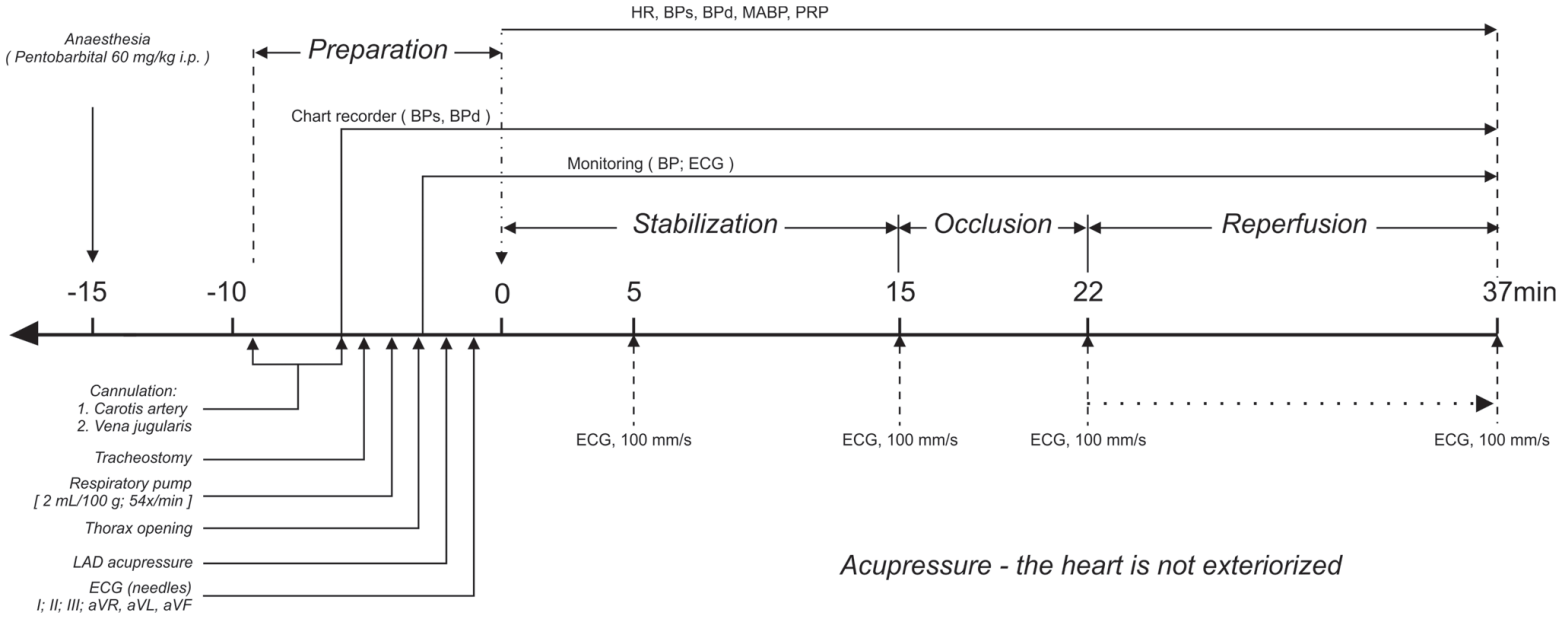
Schedule of the protocol study.

During the stabilization period (15 min) any rat with dysrhythmias or with a sustained drop in mean arterial blood pressure below 70 mmHg caused by the procedure, was discarded from the study as prescribed by the Lambeth Convention and others [8], [9], [18]. The ligature was passed through a piece of plastic tubing (2 cm long, 2 mm od, 1.2 mm id) and the LAD was occluded for 7 min by applying tension to the ligature while pressing the distal part of the plastic tube onto the surface of the heart. Tension was maintained by clamping the tube and by the ligature. Successful occlusion and ischemia were confirmed by a pronounced decrease in arterial pressure and ECG alteration (e.g. ST-elevation). Reperfusion (15 min) was initiated by removing the clamp and releasing the tension on the ligature. Reflow was confirmed by significant ECG changes (e.g. reversal of ST segment elevation) immediately upon release of the ligature as well as by injecting Evan’s blue dye (Fig. 2).

### Experimental Design

The control (0.4% DMSO), M-2, M-3 and M-8 group each consisted of 16 rats. 20 mg/kg of M-2, M-3 or M-8, or 5 mL/kg of the excipient (0.4% DMSO water solution) was given orally twice, one day and one hour (i.e. 24+1 h) before LAD occlusion.

7 min of ischemia induced by LAD occlusion followed by 15 min of reperfusion is a simple, reproducible and effective method to induce a large number of severe arrhythmias and a resulting high rate of mortality in the rat model. This allows for effective examination of the antiarrhythmic potential of the tested agents [8], [9]. Additionally, the short period of 7 min of ischemia followed by reperfusion mimics the series of events that lead to arrhythmias seen in clinical scenarios such as the sudden resolution of coronary spasm or revascularization or thrombolytic procedures [21], [22]. Arrhythmias of such etiologies are potentially fatal and are a rare but important cause of the sudden death in clinics [23], [24]. In our study animal mortality was caused by continuous, alternating ventricular fibrillation (VF) and ventricular tachycardia (VT) episodes during at least the first two minutes of reperfusion linked with a drastic drop in blood pressure (BP). Arrhythmias were qualified according to the Lambeth Conventions [18], where VT was defined as a run of four or more consecutive ventricular premature beats. An episode of VF was defined as a signal where individual QRS deflections could not be easily distinguished from each other and where rate could no longer be measured [18]. The duration of each episode was measured in seconds and the sum of these durations during 15 min of reperfusion was analyzed. Because of the short time of occlusion, the arrhythmias occurring in this period were negligible [8], [9] as it might be also seen from the characteristic BP tracing and that is why they were not taken into consideration. Because of the short duration of ischemia, there was no myocardial infarction.

The rationale for the doses used is as follows. In our previous paper in which the dose-response study with oxy metabolites was carried out [6], 20 mg/kg given orally twice (24 and 1 h before occlusion) produced optimally achievable protection in the model used, i.e. very significant anti-arrhythmic effects. Thus this dose was chosen for the present study to compare the actions of M-2, M-3 and M-8.

### Measured and Calculated Parameters

In addition, following parameters were calculated: mean arterial blood pressure (MABP; BPd +0.42×ΔBP), heart rate (HR) calculated from ECG, pressure rate product (PRP; BPs×HR/1000) as an indirect index of myocardial oxygen consumption according to [25]. The duration of spontaneously reversible VF or VT that occurred during the 15 min of reperfusion were measured (using the continuous ECG recordings) and the percentage of VF or VT appearance in the groups as well as the mortality index (MI) were calculated.

### Data Analysis and Statistical Procedures

VF and VT incidence and duration were measured only in the surviving animals. Results are expressed as mean±SEM, except the MI, VF and VT episodes. The chi-square-test (*χ*2) was used to estimate the significance between the incidence of arrhythmias and mortality in all comparisons. Due to difference in number and small number of animal in the groups and since they are not normally distributed, the *Kruskal-Wallis* test was used for comparisons. In all cases differences were considered significant if *P*<0.05.

## Results

Mortality was significantly reduced by M-2 and M-3 (*P*<0.05 in each case), with M-3 preventing mortality completely (Table 1).

**Table 1:** Effects of oral dose of the three furnidipine metabolites (M-2, M-3, M-8) on mortality and arrhythmias in the rat model for reperfusion-induced arrhythmias.

Experimental group, dose (20 mg/kg)	Number of animals	Mortality index (MI) [%]	Ventricular fibrillation (VF)		Ventricular tachycardia (VT)	
			Duration [s]	Incidence [%]	Duration [s]	Incidence [%]
Control	*n = *16	43.75	19.20±3.31	100	38.98±2.88	100
		(7/16)	(9/9)		(9/9)	
M-2	*n = *16	6.25*	6.90±1.24**	26.6***^†^	30.48±3.52^†^	100
		(1/16)	(4/15)		(15/15)	
M-3	*n* = 16	0*	0***	0***	15.58±2.47***	100
		(0/16)	(0/16)		(16/16)	
M-8	*n* = 16	31.25	6.6±1.54**	36.36**^††^	42.52±3.84^†††^	100
		(5/16)	(4/11)		(11/11)	

Data in parentheses are presented as the number of rats with MI, VT or VF/total number of rats in each group. Values are expressed as mean ± SEM. The chi-square-test (*χ*2) was used to estimate the significance between the incidence of arrhythmias and mortality in all comparisons. The *Kruskal-Wallis* test was used for others. Values marked with *(*P*<0.05), **(*P*<0.01) or ***(*P*<0.001) are significantly different from control. Values marked with ^†^ (*P*<0.05), ^††^ (*P*<0.01) or ^†††^ (*P*<0.001) are significantly different from M-3 group.

Each of tested metabolite caused a decrease in incidence and duration of VF. When compared to control M-2 and M-3 caused the most significant decrease in VF incidence (*P*<0.001) with M-3 entirely protected against VF occurrence, while the M-8 reduced this parameter less significantly (*P*<0.01). VF duration, estimated in survived rats only, was significantly reduced by M-2 (*P*<0.001) and M-8 (*P*<0.01) in comparison to control and M-3 (Table 1).

None of the three metabolites diminished VT incidence. Comparing to control, only M-3 significantly decreased VT duration (*P*<0.001) and it was significantly greater than M-2 (*P*<0.05) and M-8 (*P*<0.001) (Table 1).

In stabilization period MABP was significantly lowered by M-2 (*P*<0.05). During the occlusion and reperfusion M-8 caused a significant decrease of MABP and this effect was marked to control, M-2 and M-3 (*P*<0.05).

**Figure 3:**
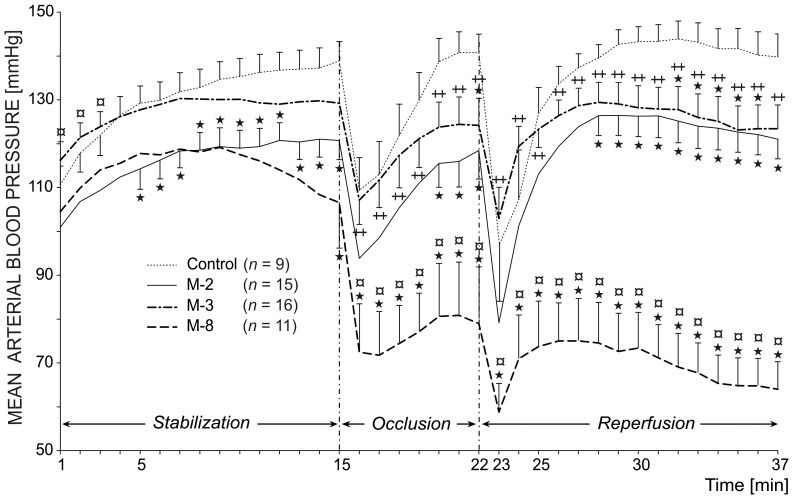
Effects of oral administration (20 mg/kg; 24+1 h before occlusion) of the tested three metabolites of furnidipines’ (M-2, M-3, M-8) on mean arterial blood pressure in the rat model for ischemia- and reperfusion-induced arrhythmias. Data are expressed as mean ± SEM. For sake of simplicity, SEM values are depicted by the vertical lines in the entire trace of control but in the traces of treated groups only when values achieved significance. Values marked with *(*P*<0.05) are significantly different from control. Values marked with ^¤^(*P*<0.05) are significantly different from M-2 and values marked with ^++^(*P*<0.05) are significantly different from M-8.

Only M-3 markedly prevented the steep drop in MABP just after reperfusion (22–23^rd^ min) seen in control and M-8 treated rats (*P*<0.05). At the end of reperfusion all tested metabolites (M-2, M-3 and M-8) significantly lowered MABP as compared to control rats (*P*<0.05) (Fig. 3). Analogically to MABP, PRP values were significantly lowered in M-2 treated rats (*P*<0.05). During the occlusion and reperfusion M-8 caused a significant decrease of PRP and this effect was marked to control, M-2 and M-3 (*P*<0.05). Only M-3 markedly increased PRP just after reperfusion (22–23^rd^ min) seen in control and M-8 treated rats (*P*<0.05). At the end of reperfusion all tested metabolites (M-2, M-3 and M-8) significantly lowered PRP as compared to control rats (*P*<0.05) (data not shown).

None of the three metabolites had any relevant influence on the HR throughout the experiment (data not shown).

## Discussion

In previous study conducted by us, we noted that FUR and NIF given orally and i.v. had comparable anti-arrhythmic effects [6]. The anti-arrhythmic action of NIF after its i.v. administration was also confirmed in experiments conducted by others [26], [27]. Furthermore, we have proved that FUR could be effective as a cardio-protective agent mainly due to limiting the incidence of lethal arrhythmias and in consequence preventing rats mortality [12], [28]. This led us to the conclusion that it could be reached through pathways other than only L-type calcium channel blocking. Therefore we tested oxy dihydropiridines and we revealed that the cardio-protective and anti-arrhythmic effect achieved after their administration is highly better than the parent DHP [6]. Subsequently, we presumed that its worth to study metabolites of FUR and to check their influence on cardiovascular system.

Our findings reveal beneficial influence on heart tissue and vessels in rats treated with M-3 and M-2. In these rats a significant reduction in mortality and VF incidence and duration were observed.

There was also contrastingly different influence of all three metabolites (M-2, M-3, M-8) on MABP and PRP. While the rats were strongly hypotensive in the M-8 treated group, the MABP unchanged after M-2 administration and significantly increased during first minutes in reperfusion after M-3. The PRP trace was nearly the same as MABP.

With respect to arrhythmias and MABP analogical observations were made with oxy nifedipine [6]. It must be mentioned that oxy nifedipine is metabolized to a final product – M-2 and that means that M-2 is not only a final metabolite of FUR, but also of nifedipine. Hence, M-2, as a common molecule to NIF and FUR, evokes the most beneficial effects on heart tissue, what we previously proved [13], [14]. Few years earlier others proved that NIF evoke vasodilatation of coronary artery through NO mediated process in dogs [29]. Based on these observation, we speculate that the significant activity of M-2 which demonstrates in cardio-protective profile with independent and more pronounced action than their parent drug might be caused by NO pathway [13], [14], [28]. Moreover, through the NO mediated process M-2 could evoke wide range of biological properties e.g. maintain vascular homeostasis, regulate the local cell growth and protect the vessel from injurious in consequences of platelets and cells circulating in blood. Subsequently, all these effects strongly suggest its anti-oxidant properties. The another possible mechanism of action of DHPs’ metabolites is not still fully elucidated. Some authors predicate that M-2 could be a molecule acting on ATP sensitive outward potassium channel, however there are suggestions that activity through these channels might exert malignant ventricular arrhythmias [30]–[34]. Nevertheless, ATP sensitive potassium channels are likely target. All these mentioned above arguments brought us to the conclusion that the effectiveness of FUR and NIF in arrhythmias and hypertension is due to their metabolite – M-2. This novel observation give an impulse for further investigations into the molecular mechanisms (including NO release and potassium channels activity), clinical implications and potential role of these therapeutics. However, the evaluation of the pharmacological targets responsible for anti-arrhythmic actions and influence on MABP and PRP evoked by the DHPs’ metabolites was not conducted at this time. Therefore, it could be assumed that M-2 do not only possess calcium channel blocking activities, NO mediated pathway or potassium channel activity but it is also a potentially promising cardio-protective agent representing a new structural class of drugs.

It is well known that the interindividual variability of the plasma levels and the kinetics parameters of DHPs are rather large. It has been shown that the intersubject variability of the disposition of e.g. nifedipine may be due to a polymorphism of the metabolizing enzymes in liver leading to differences in the first-pass metabolism. Clearly, this main metabolite (M-2) of some “privileged structures” (i.e. having 1,4-dihydropyridine nucleus), which is rapidly and differently converted from parent drugs, at the same time being a final and stable active agent, is responsible for the most cardiovascular effects. However, these effects are modulated in part by the action of other metabolites released thought the cascade of dynamic metabolism of DHPs. It depends from their proportional presence in plasma and this is multifactorial process strongly related to environmental, local conditions (e.g. pH).

The versatility of these pharmacophore was illustrated by their actions at other ion channels, but also by their potent and selective actions at other receptor systems, including at least three receptors of the G-protein class. Apparently, the relationship among series of DHPs is rather qualitative than quantitative and the significant differences in cardiovascular profile may exist.

In fact, because of the parent drug little plasma concentration it does not play the leading role in this concert of cardiovascular effects. This is also why it could be concluded that 1,4-dihydropyridines exerts plejotropic effects, and some DHPs metabolites are much more easy to monitoring them to achieve the well-defined clinical target.

In conclusion, the results of our study provide novel evidence of the anti-arrhythmic activity of furnidipines’ metabolites in attenuating arrhythmias that were in our model induced by ischemia and reperfusion. The differential influence on MABP could indicate its use in various stage of blood pressure in patients (e.g. in hypotension – M-3, hypertension – M-8). It seems that M-2, M-3 and M-8 might find a place not only as an anti-arrhythmic therapeutic but also as a blood pressure regulated molecules and cardio-protective agents.
