# Correction: Differential Effects of Furnidipines’ Metabolites on Reperfusion-Induced Arrhythmias in Rats In Vivo

**DOI:** 10.1371/journal.pone.0114194

**Published:** 2014-11-20

**Authors:** The PLOS ONE Staff

There are errors in the Author Contributions. The correct contributions are: Conceived and designed the experiments: TFK MP. Performed the experiments: MP. Analyzed the data: KAM. Contributed reagents/materials/analysis tools: KAM MP TFK. Wrote the paper: KAM TFK.
